# Synchronous Adenocarcinoma Stomach With Marginal Zone Lymphoma: A Sporadic Occurrence and Review of Literature

**DOI:** 10.7759/cureus.41631

**Published:** 2023-07-10

**Authors:** Amitabh Upadhyay, Shashank Shekhar, Vanita Pandey, Aaditya Prakash, Kaushik Saha

**Affiliations:** 1 Oncology, Tata Main Hospital, Jamshedpur, IND; 2 Medical Oncology, Meherbai Tata Memorial Hospital, Jamshedpur, IND; 3 Pathology, Meherbai Tata Memorial Hospital, Jamshedpur, IND; 4 Radiation Oncology, Tata Main Hospital, Jamshedpur, IND; 5 Specialist, Pathology, Tata Main Hospital, Jamshedpur, IND

**Keywords:** smpc, lymphoma, gastric, stomach, synchronous

## Abstract

Synchronous adenocarcinoma of the stomach with lymphoma is extremely rare. We report a case of a 65-year-old male patient with synchronous adenocarcinoma of the stomach with nodal marginal zone lymphoma. Initial endoscopic biopsy suggested invasive moderately differentiated adenocarcinoma and a locoregional disease, per contrast-enhanced computed tomography (CECT) scans. The patient was started on neo-adjuvant chemotherapy with the 5FU, leucovorin, oxaliplatin, docetaxel (FLOT) regime and, after response evaluation, underwent radical gastrectomy. Histopathology and immunohistochemistry suggested synchronous adenocarcinoma of the stomach with marginal zone lymphoma in perigastric lymph nodes. This case is probably the first such synchronous malignancy case reported from India. The prognosis of multiple primary malignancies is usually poor because no standard guidelines are available regarding optimum treatment and sequencing of available treatment modalities. The frequency of synchronous primary cancers has been increasing in recent years, probably due to better diagnostic modalities, and second primary in patients with cancer should be considered as one of the differential diagnoses.

## Introduction

Carcinoma stomach was the world's fifth most common cancer in 2020, with approximately 1.1 million new cases, and is the fourth leading cause of cancer death, with around 800,000 deaths [1]. The median age of diagnosis for carcinoma stomach is approximately sixty-eight years, and the frequency in males is about double that in females [2]. The highest incidence rates for carcinoma stomach were observed in Eastern Asian males, with 32.5 per 100,000 population. Male populations of Japan (48.1), Mongolia (47.2), and Korea (39.7) had the highest incidence in the world, while it was lowest in Africa with incidence rates < 5 per 100,000 [1,2,3]. Approximately 75% of new gastric cancer cases and deaths are reported in Asia [1,2,3]. The epidemiological studies have predicted the annual burden of carcinoma stomach to be about 1.8 million new cases and 1.3 million deaths by 2040 [3]. The estimated five-year survival for gastric cancers is 20- 30%, with the recent increase in survival noted probably due to newer drugs and advanced treatment options [1-4]. 

Non-Hodgkins lymphoma (NHL) is another common malignancy, and in 2020, an estimated 5.4 lakh new patients of NHL were diagnosed worldwide, and approximately 2.6 lakh patients died from the disease [5]. The gastrointestinal (GI) tract is a common site for extranodal lymphoma, and abdominal lymph nodes involvement in lymphoma is common. Diffuse large B cell lymphoma (DLBCL) is the most common GI lymphoma, followed by low-grade marginal zone/mucosa associated lymphoid tissue (MALT) lymphomas [6]. 

Synchronous multiple primary cancer (SMPC) is diagnosed together or within six months of another cancer diagnosis, as defined by Warren and Gates's criteria [7]. According to the criteria, both neoplasms must be malignant, the two neoplasms must be anatomically separate, and the possibility of the second primary neoplasm being a metastasis from the index tumor must be excluded [7]. SMPC is an infrequent occurrence and is always a diagnostic and therapeutic dilemma for a clinician. Here we report a rare case of SMPC from India. 

## Case presentation

**Figure 1:**
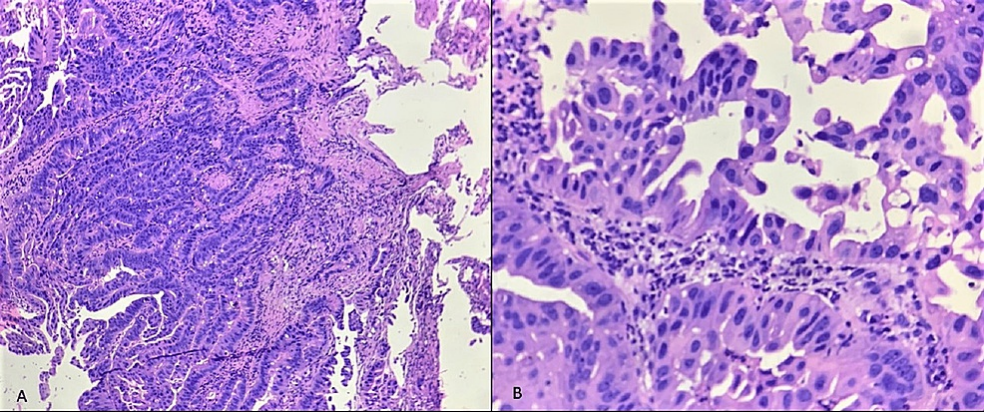
1A: Gastric biopsy Haematoxylin and Eosin (H&E) staining in 10X magnification showing malignant cells arranged in acini, cords invading the stroma, 1B: H&E 40X magnification showing malignant cells with an elongated hyperchromatic nucleus and scant cytoplasm, S/O Intestinal type adenocarcinoma

A 65-year-old male patient with an Eastern Cooperative Oncology Group (ECOG) performance status of one came to our hospital complaining of malaise, poor appetite, melena, and occasional haematemesis for two months. The patient was a tobacco chewer for the last 20 years and had no significant family history. An initial esophagogastroduodenoscopy (EGD) showed a large friable, ulcerated lesion with central necrosis involving the gastric fundus and body, suspicious of carcinoma stomach. An abdominal contrast-enhanced computed tomography (CECT) scan revealed irregular and heterogenous enhancing wall thickening (max 2.5 cm) involving the gastroesophageal (GE) junction, fundus, and body of the stomach, with extension to the peri gastric fat with peri gastric fat stranding and enlarged peri gastric nodes, most significant 15 mm in size, s/o neoplastic lesions. There was no evidence of any metastasis in the CECT of the thorax. EGD biopsy suggested invasive moderately differentiated adenocarcinoma (Figure 1), and a rapid urease test for H. pylori was negative. The patient received neo-adjuvant chemotherapy (NACT) with the 5FU, leucovorin, oxaliplatin, docetaxel (FLOT) regime with standard antiemesis protocol, including dexamethasone.

**Figure 2:**
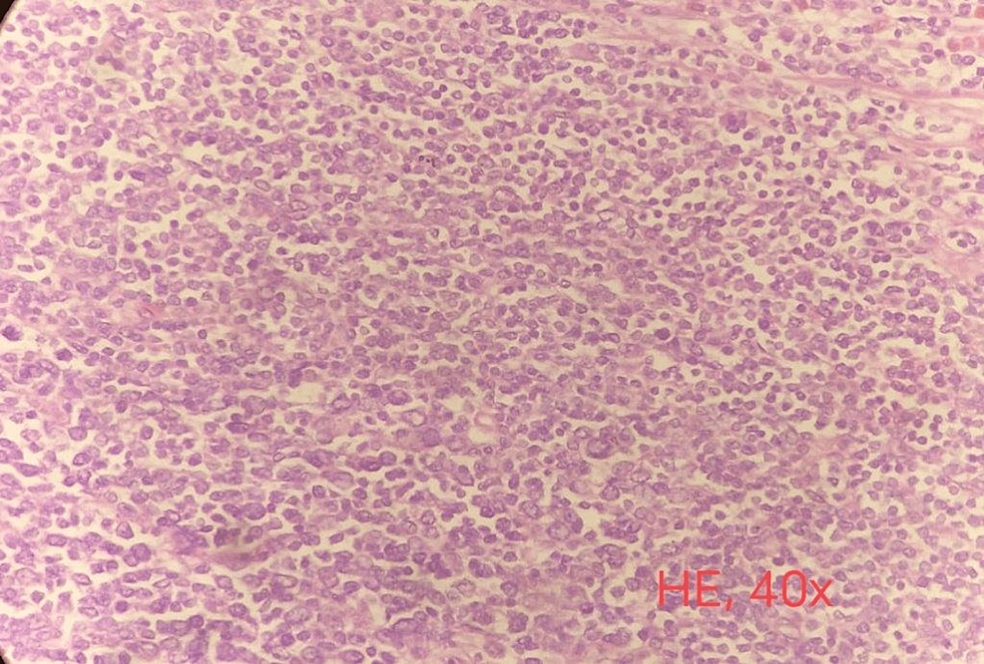
Perigastric lymph nodes with Haematoxylin and Eosin (H&E) staining in 40X magnification, showing architectural effacement of lymph node with replacement by the population of monotonous small to medium-sized lymphoid cells

**Figure 3:**
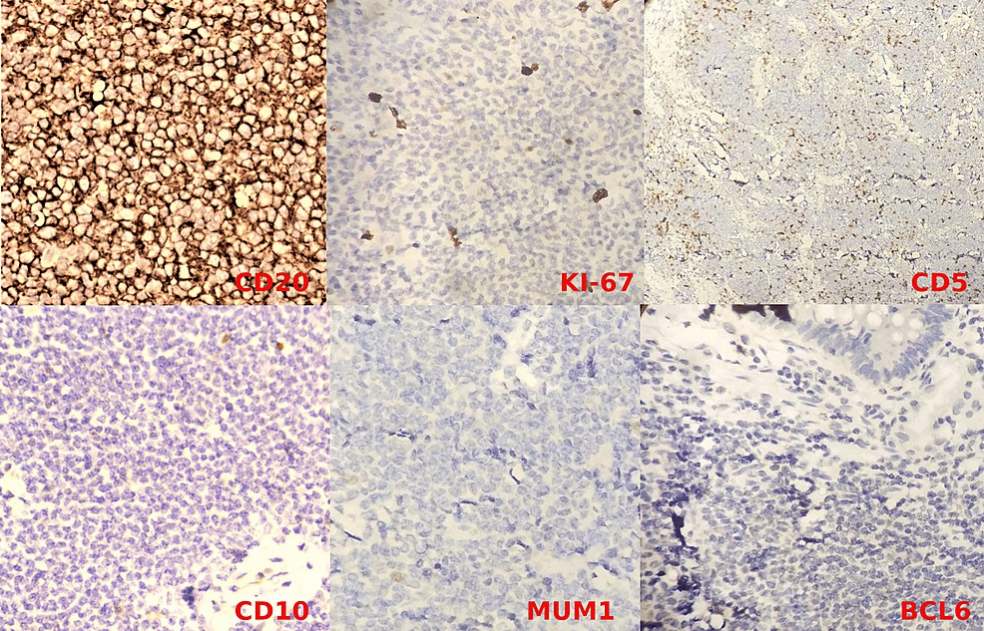
IHC on perigastric lymph nodes showing CD 20 strongly positive, while negative for CD5, CD 10, MUM-1, Bcl-6 with Ki-67 value 05%

After completing four cycles, response evaluation with an abdominal CECT suggested a partial response. Similar to the previous scan, asymmetrical wall thickening was still noted but limited in the fundus and GE junction region. There was a significant interval decrease in the maximum thickness of the wall, which was 7.8 mm in the part of the fundus vs. 2.5 cm in the previous scan. The wall thickness got resolved entirely in the body of the stomach compared to the last scan. The fat plane of the stomach with the adjacent hepatic parenchyma was well maintained. The perigastric enlarged lymph nodes noted in the previous scan were entirely resolved. The patient underwent radical gastrectomy & splenectomy as the splenic hilum appeared to be infiltrated intraoperatively. Histopathological examination suggested spleen negative for granuloma or malignancy. The stomach had residual moderately differentiated adenocarcinoma, a papillary variant. Tumour was located in the lesser curvature, fundus region, and multiple; the largest tumor measured 3.0 cm in the most significant dimension and invaded the serosa. All margins (proximal, including oesophageal doughnut, distal, and radial margins) were tumor-free. The tumor was 4.5 cm from the oesophageal resection margin (closest margin). Perigastric lymph nodes showed architectural effacement with replacement by the population of monotonous small to medium-sized lymphoid cells, strongly favoring low-grade lymphoproliferative disease (Figure 2). Immunohistochemistry (IHC) on perigastric lymph nodes showed CD 20 strongly positive, Bcl-2 positive, while negative for CD3, CD5, CD10, Bcl-6, and MUM-1 in lesional cells. Ki-67 was 05% suggestive of low-grade B cell non-Hodgkin's lymphoma favoring marginal zone lymphoma (Figure 3). 

**Figure 4:**
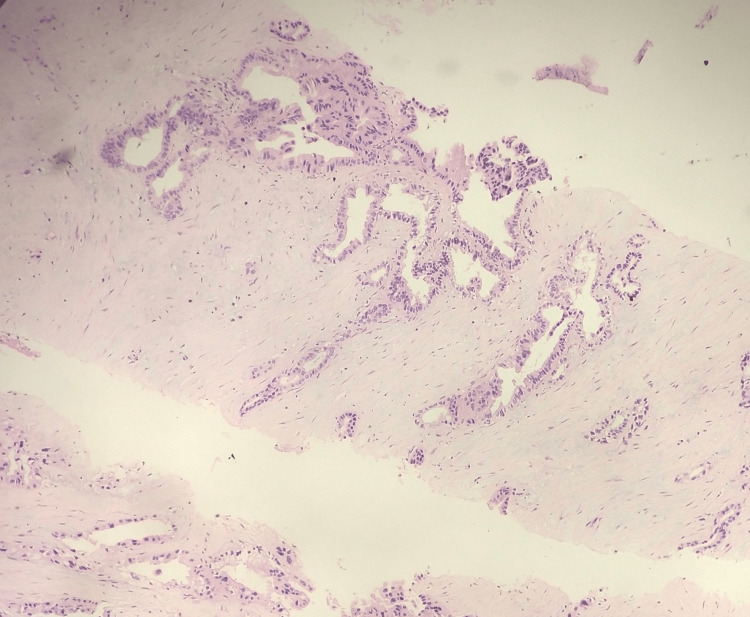
Section from linear cores of the liver with H&E, 40X magnification showing infiltrative glands of varying sizes in a crowded arrangement, lined by atypical cells, set against desmoplastic stroma.

**Figure 5:**
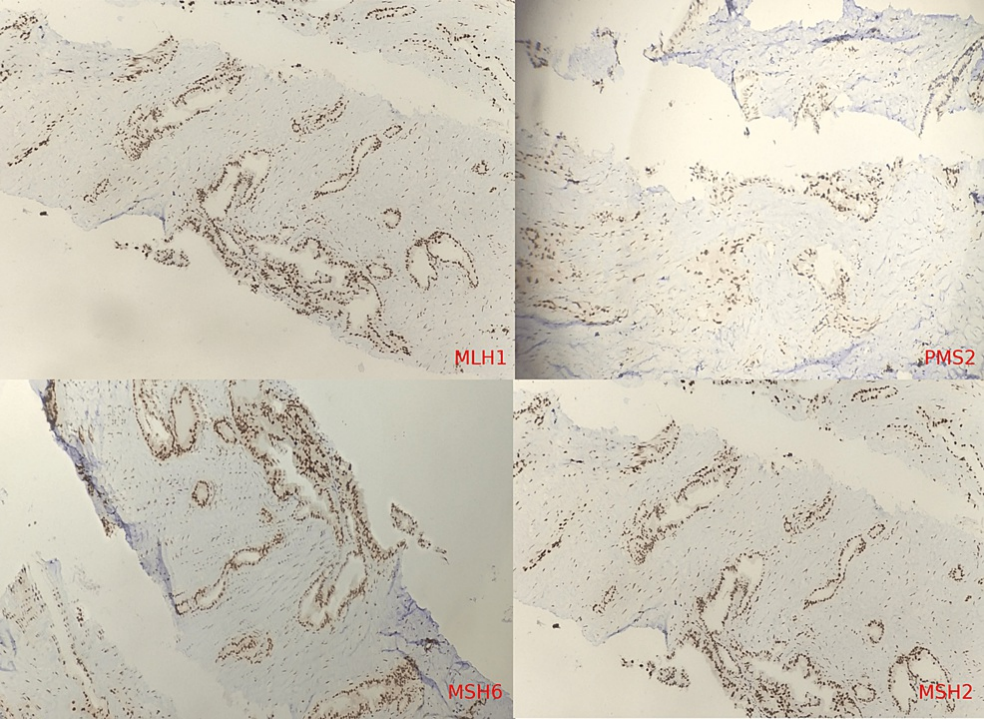
IHC for Mismatch Repair proteins (MMR) showing retained expression for MLH1, PMS2, MSH6, and MSH2

We discussed the case in our institutional tumor board, and subsequently, the patient completed four cycles of adjuvant chemotherapy with the FLOT regime and necessary vaccinations post-splenectomy. There was no evidence of any lymphomatous involvement in bone marrow aspiration & biopsy performed after surgery. The patient was advised for involved site radiotherapy (ISRT) with potential benefits in NHL as well as adenocarcinoma stomach, but the patient defaulted. The patient came after four months with CECT abdomen and thorax showing interval appearance of the non-enhancing lesion with enhancing peripheral margins in liver segment VIII, size 5.6x3.4x2.9 cm, subcentimeter-sized enhancing mesenteric and retroperitoneal nodes. Ultrasonogram (USG) guided biopsy from the liver nodule was suggestive of metastatic moderately differentiated adenocarcinoma (Figure 4). IHC for Her2/neu was positive [3+] and negative for mismatch repair proteins (MMR), showing retained expression for MLH1, PMS2, MSH6, and MSH2 (Figure 5) on liver biopsy. The patient was started on palliative chemotherapy with the 5FU, leucovorin, irinotecan (FOLFIRI) regime. The patient was not affordable to take anti-HER2 therapy, ramucirumab, or immunotherapy. 

## Discussion

The synchronous occurrence of both gastric adenocarcinoma and lymphoma is sporadic, and little is known regarding the clinicopathologic characteristics of such cases. Helicobacter pylori (H. pylori) is believed to play a causative role in chronic active gastritis, peptic ulcers, and gastric malignant neoplasms, including adenocarcinoma and lymphomas [8,9]. H. pylori is associated with about 70% of gastric cancers. The possible mechanism for H. pylori as an etiological agent for gastric lymphoma is chronic infection leading to inflammation with hormonal and cellular changes leading to clonal expansion of B cells [8,9]. Conversely, Epstein-Barr virus (EBV) has also been implicated in the pathogenesis of various types of malignant lymphomas and carcinomas arising in several organs, including the stomach [9]. Approx. 10% of gastric adenocarcinomas and 9% of gastrointestinal tract (GIT) lymphomas are associated with EBV positivity [10]. 

A total of 56 cases of synchronous adenocarcinoma, stomach, and lymphomas were reviewed by Hamaloglu et al. in 2006 [11]. Similarly, 57 patients were reported in a review of such cases by Namikawa et al. for a study period from 1990 to 2013 in 2014 [12]. Subsequently, similar individual cases were reported by Weng CH et al. in 2016 [13] and Meng et al. in 2019 [14]. Synchronous primary gastric triple-hit high-grade B-cell lymphoma and gastric adenocarcinoma were reported by Claudia C et al. in 2021 [15], and a case of T-cell lymphoma and gastric adenocarcinoma was reported by Narcisa G et al. in 2021 [16]. There are five reported cases of synchronous gastric cancer and Hodgkin's lymphoma in the literature [17]. Most of the reported data is from Eastern Asian countries like Japan, China, and Korea, with sporadic publications from Western countries. The probable reason is the much higher incidence of gastric cancer in Eastern Asian countries. We tried to find a similar SMPC case report from India but could not get any. 

In our case, H Pylori was negative, and EBV testing was not done; we speculate it to be incidental synchronous adenocarcinoma of the stomach with nodal marginal zone lymphoma as we could find evidence of lymphoma only in perigastric lymph nodes. Another possibility is that the low-grade lymphoma might be present in some stomach areas and could have resolved with the FLOT chemotherapy regime. Docetaxel and oxaliplatin have excellent activity against lymphomas, which are part of the FLOT regime [18,19]. Lymphomas are very sensitive to steroids also, and a total of 36 mg of dexamethasone per cycle, amounting to 72 mg per month, was given as premedication and for post-chemotherapy emesis prophylaxis. This exceedingly uncommon occurrence of multiple synchronous primaries is challenging to manage because of the lack of defined guidelines and the dilemma regarding the correct sequence of treatment modalities. The management of lymphomas is usually chemotherapy plus minus radiation, while treatment of gastric cancers is chemotherapy followed by surgery in the early stages and palliative chemotherapy in metastatic cases. There are options for anti-HER2 therapy in tumors that are HER2 positive, and immunotherapy has a role in defective mismatch repair (dMMR) patients and cases with PDL1-positive tumors in combination with chemotherapy [20].

The treatment algorithm depends on tumor locations, stage of both tumors, symptom burden, the aggressiveness of both tumors, chemosensitivity of tumors, and performance status of the patient and usually requires multidisciplinary discussions with the involvement of surgeons, medical oncologists, pathologists, radiologists, and radiation oncologists. Usually, a chemotherapy regimen is selected for more aggressive and symptomatic tumors, and the drugs with a spectrum of activity in both tumors should be preferred wherever possible. We can even add Rituximab, a monoclonal antibody for CD20, along with chemotherapy for adenocarcinoma part, without any significant increase in toxicity with the potential of benefit in NHL and gastric cancer. The surgical modality and radiation should be appropriately sequenced after multidisciplinary discussion. The patient was started on NACT for adenocarcinoma stomach in the current case. In the radical gastrectomy specimen, lymph nodes showed the presence of low-grade lymphoma, and adenocarcinoma was limited to gastric tissues only. It was challenging to prove the synchronous occurrence of adenocarcinoma in the gastric wall and low-grade lymphoma in perigastric lymph nodes upfront, except for the patient undergoing upfront surgery and lymph node dissection but, that is not standard of care for locally advanced carcinoma stomach. This patient completed adjuvant chemotherapy with the FLOT regime. There is no guideline regarding further treatment required for the lymphoma part, as lymph nodes were removed during surgery, and there was no residual disease in post-operative scans. After our multidisciplinary discussion, we offered adjuvant radiation, which was likely to have a potential advantage in both adenocarcinoma stomach and lymphoma. However, the patient defaulted for four months and returned with a recurrence of adenocarcinoma with liver metastasis. Literature has recommended treatment focus on more deadly cancer that is carcinoma stomach than lymphoma part, and the same was followed in this case. Most of the deaths have happened due to carcinoma part only, as per literature, and the same natural history was seen in this case. 

## Conclusions

This case is probably the first synchronous adenocarcinoma stomach and NHL case reported from India. It reinforces the importance of having this rare phenomenon in mind and strong clinical acumen while dealing with such cases. This case provides a concise literature review regarding the existing literature. It adds further insight for the healthcare community towards the etiology and management of such rare occurrences. This case adds value to the current literature by reinforcing that management of these cases should focus on more aggressive histology and symptomatic tumor out of the two cancers. This case also highlights the importance of a thorough endoscopic examination of the whole stomach, GE junction, and duodenal area for any other suspicious area and prompt biopsy from other suspicious sites to rule out synchronous malignancy. 
